# Clinical Validation of a Pixon-Based Reconstruction Method Allowing a Twofold Reduction in Planar Images Time of 111In-Pentetreotide Somatostatin Receptor Scintigraphy

**DOI:** 10.3389/fmed.2017.00143

**Published:** 2017-08-30

**Authors:** Philippe Thuillier, David Bourhis, Philippe Robin, Nathalie Keromnes, Ulrike Schick, Pierre-Yves Le Roux, Véronique Kerlan, Philippe Chaumet-Riffaud, Pierre-Yves Salaün, Ronan Abgral

**Affiliations:** ^1^Department of Endocrinology, University Hospital of Brest, Brest, France; ^2^Department of Nuclear Medicine, University Hospital of Brest, Brest, France; ^3^EA GETBO 3878, IFR 148, University Hospital of Brest, Brest, France; ^4^Department of Oncology-Radiotherapy, University Hospital of Brest, Brest, France; ^5^Department of Nuclear Medicine, University Hospital of Paris-Sud Bicêtre, Le Kremlin-Bicêtre, France

**Keywords:** signal and image processing, Pixon-based method, somatostatin receptor scintigraphy, planar images, half time acquisition

## Abstract

**Objective:**

The objective of this study was to evaluate the diagnostic efficacy of Pixon-based reconstruction method on planar somatostatin receptor scintigraphy (SRS).

**Methods:**

All patients with neuroendocrine tumors (NETs) disease who were referred for SRS to our department during 1-year period from January to December 2015 were consecutively included. Three nuclear physicians independently reviewed all the data sets of images which included conventional images (CI; 15 min/view) and processed images (PI) obtained by reconstructing the first 450 s extracted data using Oncoflash^®^ software package. Image analysis using a 3-point rating scale for abnormal uptake of 111 Indium-DTPA-Phe-octreotide in any lesion or organ was interpreted as positive, uncertain, or negative for the evidence of NET disease. A maximum grade uptake of the radiotracer in the lesion was assessed by the Krenning scale method. The results of image interpretation by the two methods were considered significantly discordant when the difference in organ involvement assessment was negative vs. positive or in lesion uptake was ≥2 grades. Agreement between the results of two methods and by different scan observers was evaluated using Cohen κ coefficients.

**Results:**

There was no significant (*p* = 0.403) correlation between data acquisition protocol and quality image. The rates of significant discrepancies for exam interpretation and organs involvement assessment were 2.8 and 2.6%, respectively. Mean κ values revealed a good agreement for concordance between CI and PI interpretation without difference of agreement for inter/intra-observer analysis.

**Conclusion:**

Our results suggest the feasibility to use a Pixon-based reconstruction method for SRS planar images allowing a twofold reduction of acquisition time and without significant alteration of image quality or on image interpretation.

## Introduction

Neuroendocrine tumors (NETs) are a group of tumors of common definitions and characteristics originating in tissues that contain cells derived from the embryonic neural crest and representing nearly 1% of all neoplasia (1). Moreover, the annual incidence rate of NETs increased during the last 30 years and is actually estimated to be roughly 5 cases per 100.000 (2, 3). Thus, NETs can arise in many locations (mainly in gastrointestinal system) and often secrete various hormones, which are responsible for different clinical manifestations in 20–30% of cases, classified as functional tumors. Histopathological classifications divided them into well-differentiated (WD) NETs including grades 1 (G1) and 2 (G2) that are the most frequent presentation, and grade 3 poorly differentiated carcinomas whose prognosis is pejorative (4–6).

Functional imaging plays a major role in the characterization of NETs including staging, therapeutic management, and recurrence diagnosis and treatment decision for peptide receptor radionuclide therapy (PRRT) that depends on the histological differentiation (7–10). Somatostatin receptor scintigraphy (SRS) with 111In-Pentetreotide is recommended for WD-NETs because this radioactive tracer derives from a somatostatin analog that binds preferentially its subtype 2 and 5 receptors, usually expressed on the tumor cell surface of G1–G2 forms. Diagnostic performances of SRS are excellent (11) for WD-NETs: a review conducted on 35 centers and including approximately 1,200 patients showed a median sensitivity of 84% to detect tumors (12).

European guidelines for imaging procedures of SRS recommend two sets of planar acquisition with at least one single-photon emission computerized tomography (SPECT) acquisition ± coregistered with tomodensitometry (CT) for attenuation correction and to improve localization of expressing lesions (13). These recommendations preconize to perform preferably planar and SPECT studies 24 h after injection of the radiopharmaceutical because of low background activity, decreasing the risk of missing lesions expressing a rather low density of somatostatin receptors. Moreover, despite a relatively high background radioactivity, 4-h planar images have the advantage of negligible bowel activity and are also required. So, planar scintigraphy images remain essential for SRS interpretation, providing to the nuclear medicine physician temporal resolution information. Indeed, the comparison of early and late planar scintigraphy images enables to better differentiate physiological digestive and pathological tumor uptakes (14). Furthermore, two times SPECT–CT acquisition could not be performed for dosimetric concerns in head, neck, chest, abdomen, and pelvis area despite its diagnostic performance is probably better (15, 16). On the contrary, planar scintigraphy images are key tools to choose the target area where SPECT–CT acquisition will be performed. But, each planar scintigraphy series includes three anterior–posterior fields of view that last finally 1½ h for patient, without counting the time acquisition of complementary SPECT–CT. Although required, planar scintigraphy acquisitions are also constraining, and it is a time-consuming process for the patient and represents an economic overload due to the duration of use of gamma cameras (opportunity costs).

The Pixon method is an adaptative image processing, initially used for astronomical observations (17, 18) and allowing a smoothing spatially correlated to signal variations without loss of resolution (19). This method has initially been studied for reconstruction of different scintigraphy planar images, especially in 99mTc-methylene diphosphonate, 67Ga-citrate, 123I-metaiodobenzylguanidine (123I-MIBG), and 99mTc-dimercaptosuccinic-acid functional imaging (20, 21); the method showed a significant increase in the signal-to-noise ratio (SNR) suggesting a reduction in image acquisition time and in the administered radioactivity of the radio-pharmaceuticals. Due to an unusual appearance of pure Pixon images that could disturb the nuclear physician interpretation (20), the OncoFlash^®^ reconstruction software package (Siemens Medical Solutions^®^, Erlangen, Germany) has recently been developed. This reconstruction processing consists to linearly blend the pure Pixon image with the unprocessed image while maintaining an equivalent SNR. This type of blending can reduce planar scintigraphy duration or injected dose by 50% in different types of planar scintigraphy without impact on the clinical value as shown by previous studies (22–24).

To the best of our knowledge, no study investigating the diagnostic utility of Pixon-based method for reconstruction of 111-In-SRS planar images has been reported.

Therefore, the objective of the present study was to validate the clinical utility of the Oncoflash ^®^ software package for reconstruction of planar SRS images. The study was based on our hypothesis that this image processing method reduces image acquisition time by twofold without any compromise on the diagnostic performance and confidence level for scan interpretation.

## Materials and Methods

### Population

All patients who were referred between January and December, 2015 to the Department of Nuclear Medicine at our Institute for undergoing SRS (as part of the routine work up in NET) were recruited consecutively. These patients met all the clinical and biochemical criteria of NET as assessed by the multi-disciplinary oncology team at our Institute. The use of Pixon-based image reconstruction method did not cause any additional constraints of time/radiation dose to the patients. Furthermore, this analysis did not interfere with the end management of the patients as the final image interpretation was performed by using the standard reconstruction method. All the patients gave a written and informed consent for the use of their images to test the software.

### Imaging Technique

Somatostatin receptor scintigraphy in all the patients was performed following an intravenous injection of about 170.0 MBq of 111-In-DTPA-Phe-octreotide (OctreoScan^®^, Mallinckrodt Medical, Petten, Netherlands) using a large field of view double-headed gamma camera (SymbiaT6^®^, Siemens Medical Solutions, Erlangen, Germany) equipped with a medium energy collimator. An adequate colonic preparation was made to remove the background gastrointestinal radioactivity. For the purpose, 30.0 g of lactulose was administered in the evening after injection and then next day in the morning before acquiring the 24-h delayed image. Patients’ preparation also included residue-free diet 3 days prior to performing SRS. Three static anterior and posterior spot views covering the head and neck, thorax, abdomen, and pelvis were acquired at 4 and 24 h (256 × 256 matrix, 15 min per view). At 24 h, SPECT/CT acquisition was also made of the area corresponding to the abnormal uptake on the planar images.

#### Conventional Images

**Figure 1:**
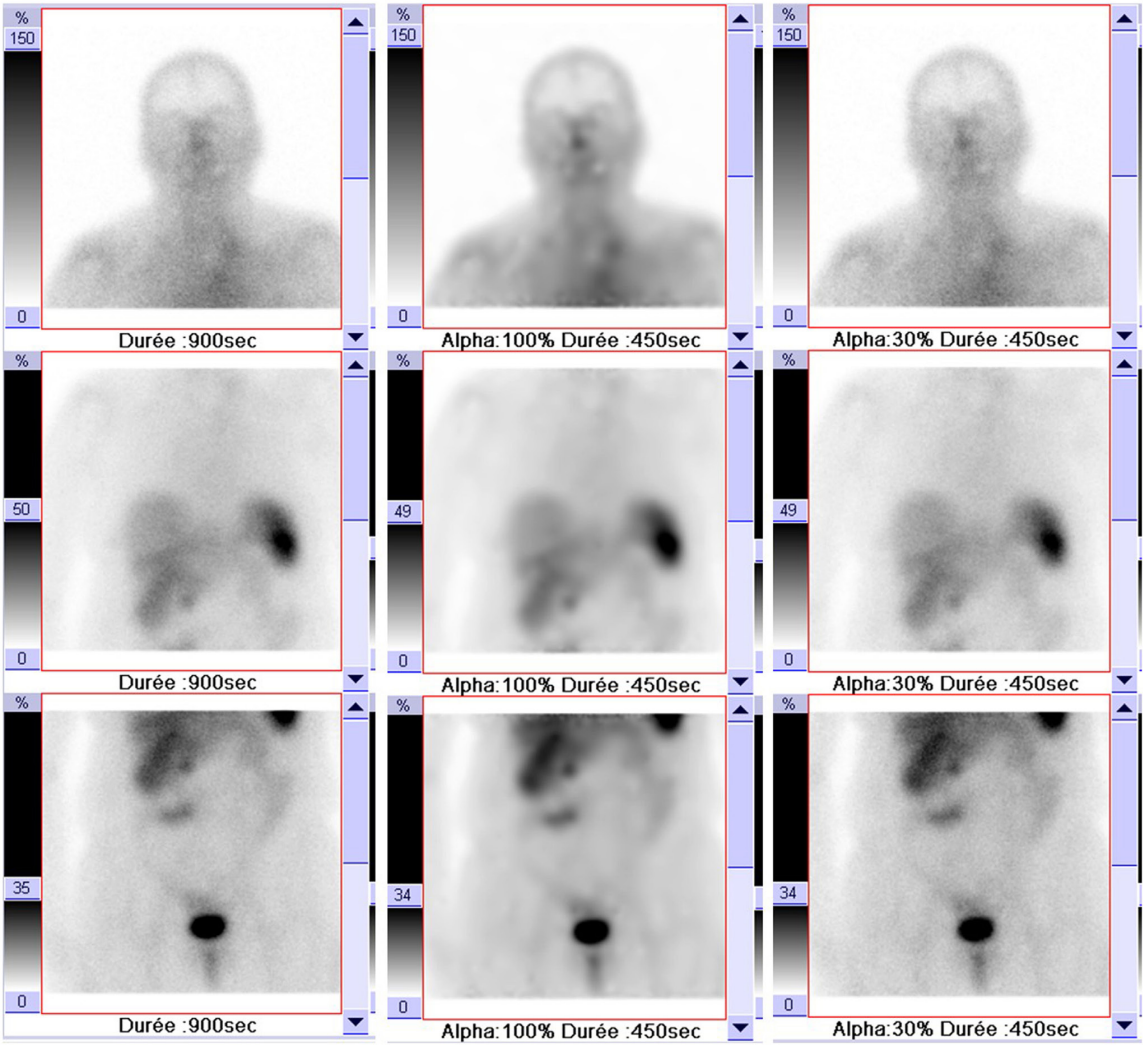
Example of a 75-year-old patient’s images with different acquisitions: conventional images (900 s; left column); processed images with 30% blending (450 s; right column), and a pure Pixon with 100% blending (450 s; middle column).

Conventional images (CI) (Figure 1) were acquired as raw image data on Syngo^®^MI application as above without processing.

#### Processed Images (PI)

Processed images (Figure 1) were obtained by reconstructing the first 450-s extracted data using Oncoflash^®^ software package with following parameters: denoising level = 1.7, maximum kernel radius = 10, number of kernel = 12, maximum iterations = 20, and a 30% blending of the pure Pixon image (Figure 1) with the unprocessed image, as suggested by Mawlawi et al. (22).

### Planar Scintigraphy Data Analysis

Three experienced nuclear medicine physicians reviewed all the images independently, and they were kept blinded to the details of patients and images. All the images (CI and PI) were anonymized with the award of a number between 1,000 and 9,999.

The images were read in two sessions, e.g., if the CI of a patient was read in first session, the corresponding PI of the patient was evaluated in the next session. The two scan reading sessions were spaced by at least a month. The duration of each session to finish reading of all the images was restricted to 1 week. To assess the intra-observer variability, another scan reading session (at 4 months later) was conducted by randomly picking up 15 (a sample size of approximately 20.0% of the total study population) images each from CI and PI series. Planar images were analyzed using a display interface on Syngovia^®^ software (Siemens Medical Solutions, Erlangen, Germany) that allowed the reader to change the image contrast.

A reading grid with several items to rate was used for image interpretation.

#### Qualitative Assessment

Based upon the visual analysis, the image quality as blurred, poor contrast, and presence of artifacts was labeled as “poor evaluation.”

#### Image Interpretation

Per-exam, per-organ, and per-lesion analyses were performed for each study.

For per-exam analysis, reader had to assess if at least one uptake was pathological on all planar images with a 3-point rating scale: positive or negative or uncertain.

For per-organ analysis, reader had to assess if at least one uptake was pathological on different organs with a 3-point rating scale: positive or negative or uncertain. Liver, lung, bones, and nodes areas (cervical, mediastinal, abdominal, and pelvic) were considered as distinct organs.

For per-lesion analysis, reader had to assess the maximal grade of uptake according to the Krenning scale as followed: grade 0, 1, 2, 3, and 4 in case of no uptake, an uptake lower than the liver, identical to the liver, above the liver, or higher than the liver or the spleen, respectively (14).

### Statistics

Statistical significance was assessed using a χ^2^ test for categorical variables and a Student’s *t*-test or Mann–Whitney test for quantitative variables.

High discordance rate, corresponding to the percentage of significant disagreement, such as negative vs. positive reading or uptake ≥2 grades evaluation between CI and PI interpretation, were calculated.

Concordance analysis between CI and PI interpretation and inter-observer/intra-observer variabilities were assessed by calculating kappa (κ) coefficient (25). According to Landis and Koch scale (26), a κ coefficient of less than 0.2 indicated poor agreement, 0.21–0.4 fair agreement, 0.41–0.6 moderate agreement (MA), 0.61–0.8 good agreement (GA), and 0.81–1 excellent agreement (EA).

All reported *p* values are two-sided. Significance level was set 0.05. Data were analyzed using SPSS statistical software v20 (IBM).

## Results

### Patients

Ninety-one SRS procedures were performed. But, only 71/91 data sets (in 59 patients; 32M: 27F; mean age = 60.2 ± 15.1 years; mean body weight = 70.2 ± 29.3 kg) were included due to the incomplete data acquisition/technical problems in the remaining 20 image series. The mean radioactivity of 111-In-DTPA-Phe-octreotide injected was 156.1 ± 29.2 MBq.

Clinical indications of SRS were: staging of histologically proven—WD-NETs in 17(24%) cases, localization of disease in situation of functional syndrome in 16(23%) cases, follow-up of patients with known disease to detect recurrence in 27(38%) cases, therapeutic response assessment in 10(14%) cases, selection of patients for PRRT in 1(1%) cases.

### Image Analysis

The three readers reviewed each of the 71 cases after conventional (71 × 3 = 213 images) and processed reconstruction (71 × 3 = 213 images), for a total of 426 interpretations. Results of agreement between CI and PI interpretation, inter- and intra-observers variabilities are, respectively, summarized in Tables 1–3.

**Table 1:** Concordance analysis between conventional image and processed images for per-exam, per-organ, and per-lesion analysis.

Analysis	*k* Values			
	1	2	3	Mean
**Per exam**				
	0.675	0.639	0.730	0.683
**Per organ**				
Lung	0.796	0.537	0.712	0.682
Liver	0.724	0.737	0.571	0.677
Abdominal area	0.706	0.619	0.808	0.711
Pelvic area	0.614	0.806	0.588	0.669
**Per lesion**				
	0.897	0.765	0.746	0.803

**Table 2:** Inter-observer variability for Conventional image (CI) and processed images (PI) for per-exam, per-organ, and per-lesion analysis.

Analysis	*k* Values	
	CI	PI
**Per exam**		
1 vs. 2	0.608	0.604
1 vs. 3	0.548	0.621
2 vs. 3	0.545	0.564
**Per organ**		
**Global**		
1 vs. 2	0.656	0.644
1 vs. 3	0.649	0.649
2 vs. 3	0.615	0.596
**Lung**		
1 vs. 2	0.695	0.777
1 vs. 3	0.796	0.640
2 vs. 3	0.553	0.552
**Liver**		
1 vs. 2	0.654	0.538
1 vs. 3	0.648	0.617
2 vs. 3	0.709	0.674
**Abdominal area**		
1 vs. 2	0.624	0.652
1 vs. 3	0.561	0.727
2 vs. 3	0.541	0.487
**Pelvic area**		
1 vs. 2	0.572	0.59
1 vs. 3	0.633	0.38
2 vs. 3	0.590	0.62
**Per lesion**		
1 vs. 2	0.682	0.713
1 vs. 3	0.795	0.711
2 vs. 3	0.67	0.708

**Table 3:** Intra-observer variability for conventional image (CI) and processed images (PI) for per-exam, per-organ, and per-lesion analysis.

Analysis	*k* Values	
	CI	PI
**Per exam**		
1	0.764	0.792
2	0.486	0.844
3	0.589	0.692
Mean	0.613	0.776
**Per organ**		
**Lung**		
1	1	0.828
2	0	0.828
3	0.8	0.455
Mean	0.6	0.704
**Liver**		
1	0.717	0.762
2	0.727	0.885
3	0.531	0.769
Mean	0.658	0.805
**Abdominal area**		
1	1	0.318
2	0.672	0.654
3	0.896	0.826
Mean	0.856	0.6
**Pelvic area**		
1	0.423	1
2	1	0.643
3	0.805	0.643
Mean	0.743	0.762
**Per lesion**		
1	0.797	0.538
2	0.868	0.895
3	0.732	0.853
Mean	0.799	0.762

#### Acquisition Quality

Among the 426 images analyzed during the first session of reading, 81(19%) have been considered of poor quality due to excessive noise or lack of contrast. There was no significant effect of the reconstruction protocol on images quality (*p* = 0.403) or administrated activity (*p* = 0.868). High body weight (*p* = 0.001) was significantly associated with poor acquisition quality.

#### Per-Exam Analysis

In combining all set of interpreted images, the three readers have considered them as positive, uncertain, and negative in, respectively, 163(38.3%), 98(23%), and 165(38.7%) cases.

The rate of very significant discrepancies of exam interpretation between CI and PI was 2.8% (6/213). Mean κ value for concordance between CI and PI analysis was 0.683(GA).

Inter-observer analysis study revealed mean κ values of 0.567(MA) for CI and 0.596(MA) for PI.

Intra-observer analysis revealed mean κ values of 0.776(GA) for CI and 0.613(GA) for PI.

#### Per-Organ Analysis

Lung, liver, abdominal, and pelvis nodes areas have been considered as positively involved in, respectively, 6.3% (27/426), 19.5% (83/426), 23.9% (102/426), and 6.1% (26/426) of cases. Bone, cervical, and mediastinal nodes areas have been considered as positively involved in less than 5% of cases. Because a low prevalence rate could affect Cohen κ coefficients and give abnormally low values (27), results in Tables 1–3 concerned only the most cited (>5%) organs as positive (lung, liver, abdominal, and pelvic areas) by the readers.

The rates of very significant discrepancies of organ involvement evaluation between CI and PI were, respectively, 3.8% (8/213), 1.9% (4/213), 3.3% (7/213), 0.5% (1/213), 2.3% (5/213), 6.1% (13/213), and 0.5% (1/213) for bone, lung, liver, cervical, mediastinal, abdominal, and pelvis nodes areas, with an average of 2.6% (39/1,491).

For lung, liver, abdominal, and pelvis nodes areas, mean κ values for concordance between CI and PI analysis were, respectively, 0.682 (GA), 0.677 (GA), 0.711 (GA), and 0.669 (GA). For other localizations, κ values varied from −0.098 to 1.

Intra-observer analysis revealed: for lung, mean κ values of 0.6 (MA) for CI and 0.704 (GA) for PI; for liver, mean κ values of 0.658 (GA) for CI and 0.805 (EA) for PI; for abdominal nodes area, mean κ values of 0.856 (EA) for CI and 0.599 (MA) for PI; and for pelvic nodes area, mean κ values of 0.743 (GA) for CI and 0.762 (GA) for PI.

#### Per-Lesion Analysis

Maximal grade of lesion uptake was, respectively, assessed as grade 0, 1, 2, 3, and 4 in, respectively, 39.4% (168/426), 13.1% (56/426), 13.1% (56/426), 15.5% (66/426), and 17.8% (76/426) of cases.

The rate of very significant discrepancies of lesion characterization between CI and PI was 1.4% (3/213). Mean κ value for concordance between CI and PI analysis was 0.803 (GA).

Inter-observer analysis study revealed mean κ values of 0.716 (GA) for CI and 0.711 (GA) for PI.

Intra-observer analysis revealed mean κ of 0.799 (GA) for CI and 0.762 (GA) for PI.

## Discussion

This prospective study suggested the potential clinical usefulness of the Pixon-based method for the processing of both early and delayed SRS planar images. The method offered essential temporal image resolution for accurately defining the field of SPECT/CT acquisition. The Pixon method is an adaptive image processing used in astronomy and allows image smoothing that spatially correlates with signal variations without loss of image resolution (17, 19). In a previous series applying this method to 18 planar images of 99mTc-methylene diphosphonate (99mTc-MDP), 67Ga-citrate (67Ga), and 123I-MIBG exams, Wesolowski et al. showed that SNR was increased with a noise reduction factor varying from 6.8-fold to 11.8-fold and suggested that it may reduce acquisition time and administered radiotracer dose significantly (20). In this study, no significant correlation (*p* = 0.403) was observed between image quality and reconstruction protocol when the image acquisition time was reduced by a factor of 2.

In a recent series of 20 whole body bone scintigraphy scans acquired with half of the standard scan-time, Ardenfors et al., however, suggested that a Pixon-based reconstruction method did not fully compensate for the loss of counts but nevertheless provided sufficient clinical information (28). The previous study by Hsiao et al. used this hypothesis to compare image quality and diagnostic accuracy in PI vs. CI to determine the minimum dose of 99mTc-MAG3 needed to perform dynamic renal scintigraphy in the pediatric population without loss of diagnostic quality or significant alteration of renal function quantification. They showed that a dose reduction ≤70% did not compromise image quality and a dose reduction of 80% induced a slight but significant decrease (*p* = 0.0074), which could be resolved with noise reduction process (23). These results are interesting and may have clinical implications in terms of a significant absorbed radiation doses’ reduction in patients undergoing nuclear medicine investigations. The major concern associated with the reduction in the administered radioactivity is the SRS images quality suitable for accurate interpretation by the physician. It has been reported that acquiring 48-h delayed images could be advantageous as these images have better image contrast due to the poor background bowel activity (13). That is why we preferred to assess a twofold reduction in acquisition time for our study. Moreover, the reduction in acquisition time improves patient comfort and limits the risk of motion artifacts.

The software used in this study has recently been developed by a medical imaging system manufacturer. This software linearly blends the pure Pixon image with the raw image while maintaining an equivalent SNR. In fact, Wesolowski et al. have suggested that the unusual appearance of a pure Pixon image could disturb physician interpretation (20). Based upon the reference of the previous study, we used a 30.0% blending with Oncoflash^®^ software to process planar images (24). These authors used a 5-point scale for the evaluation of the diagnostic performance and readers’ confidence in scan interpretation. In this study, 12 nuclear physicians participated and analyzed 39 planar images, including 12 bone scans processed by different methods (24).

Moreover, in their series evaluating 24 planar 99mTc-MDP scintiscans, Mawlawi et al. reported that a 30.0% Pixon blending provided diagnostic performance and confidence in scan interpretation that was equal to or better than the original image in 91.0 and 83.0% of the cases, respectively (22). These results were better than those obtained by using 50.0% of blending (22).

To the best of our knowledge, this is the first study focusing to the usefulness of a Pixon-based method to process planar Indium-111-DTPA-Phe1-octreotide images in clinical assessment of WD-NETs.

In the present study, Cohen κ coefficient analysis indicated a GA (0.639–0.730) for results interpreted by different scan viewers. On the other hand, a significant disagreement was observed in only 6/213 (2.8%) of the image data sets. In per-organ analysis, κ values nearly always indicated a good or an EA (0.614–0.808) excepted in lung area for reader 2, in liver and in pelvic area for reader 3, for which a MA have been reported but could be explained by the shortest experience of these two observers. In per-lesion analysis, our results revealed a good or an EA to evaluate intensity of uptake according to Krenning scale that has a real prognostic and therapeutic impact in clinical practice. In fact, the effectiveness of somatostatin analog treatment is positively correlated with lesions uptake grade in SRS (14). Moreover, high 3 and 4 uptake grades in SRS are useful for selecting potential responder patients to PRRT (29).

Concerning, inter-observer variability, we found a MA (0.564–0.621) among the scan findings of three different image readers. Moreover, the mean κ value observed did not differ (0.596 vs. 0.567) between CI and PI data. However, our data are consistent with another publication assessing the inter-observer agreement for both SRS planar images and SPECT–CT acquisitions performed in 35 patients with suspected or histologically proven NET. Apostolova et al. also found also, kappa values of 0.593 and 0.860 in planar images and SPECT–CT interpretation respectively (30). Furthermore, in considering abdominal area analysis, which is most likely site of primary disease, we found a higher average agreement between readers (GA vs. MA) in PI analysis. Otherwise, other mean κ values showed identical global agreement in per-organ inter-observer analysis. As we previously hypothesized, these results suggested the non-inferiority of PI interpretation by different physicians (in comparison with CI) to target areas where have to be performed an additional SPECT–CT acquisition, which improve diagnostic performance of exam.

Concerning intra-observer variability, our results suggested a higher global agreement in per-exam analysis for PI vs. CI interpretation, with respective mean kappa index of 0.776 vs. 0.613. Moreover, per-organ analysis showed comparable average κ values except for liver and abdominal area which showed opposite results; thus, intra-observer reproducibility in PI interpretation appeared better in liver (0.805 vs. 0.658) but lower in abdominal area (0.856 vs. 0.6). These differences could be explained by the difficulty for the physicians to distinguish whether a lesion correspond to a liver or an abdominal location in a planar image. But, as already underlined, these findings would have no consequence to select the region of interest for additional SPECT–CT acquisition. For per-lesion analysis, intra-observer variability was globally comparable in PI and CI interpretation. Interestingly, image interpretation by experienced readers showed disagreement only in one image data (PI and CI) set. However, detailed results did not highlight any significant discrepancies (≥2 grades) in the assessment of the maximal lesion uptake according to Krenning scale. These different results in per-exam, per-organ and per-lesion intra-observer analysis also suggested a good reproducibility in planar PI interpretation, at least equivalent with CI study.

Bones, cervical, and mediastinal nodes areas have been considered as positively involved by at least 1 of the 3 different observers in only less than 5.0% of the cases. These cases were thus taken into consideration for κ statistical analysis. It has been reported previously that disease prevalence could affect Cohen κ statistics and extremely low κ values could be observed (27). Our findings are in consonance with the results of this study.

In assessing different readings of our three observers, bones, cervical, and mediastinal nodes areas have always been considered as positively involved in less than 5% of cases. This was the reason why we did not take into account the κ statistics results in these locations for both inter- and intra-observer analysis. We also intentionally did not present them in our results Tables 2 and 3. In fact, Feinstein et al. reported in a previous study that prevalence of disease could affect Cohen κ statistics. Indeed, authors emphasized that in case of low prevalence (less than 10%) abnormally low Kappa index values could be observed (27), as well as in our study (varying from negative values to 1). Moreover, our findings are in accordance with a known low prevalence of metastatic involvement in bones, cervical, and mediastinal nodes areas. Lombard et al. reported that in a cohort of 668 NET patients, the observed incidence of skeletal metastases is only 6.4% and these results are in agreement with the observations in our study (31). Furthermore, in a series of 161 patients with NETs, Wang et al. confirmed histologically 8.7% of cervical and/or upper mediastinal lymph node involvement suspected by a positive SRS scan (32).

The first limitation of the study was the single center design which may induce a bias in scan interpretation as the junior readers involved in the study were all trained by the same senior consultant. Second, the receiver operating characteristic curve was not used in the present comparative study that, however, was not the aim of the study. Further, it was not possible to have the accurate gold standard lesions’ analysis, which most often is the invasive biopsy, and the histopathology analysis. Third, our Cohen κ index comparisons between CI and PI in inter- and intra-observer analysis were only descriptive. Finally, we are aware that 68Gallium (68Ga) somatostatine receptor positron emission tomography (PET) imaging should become the reference in characterization of WD-NETs and replace in the future SRS but its use is actually limited to few PET centers with 68Ga generator.

## Conclusion

In conclusion, our study shows the possibility to use in clinical routine a Pixon-based method to process 4 and 24 h planar images of 111In-SRS, allowing a twofold reduction in acquisition time without significant alteration of image quality or major impact on reader confidence.

## Author Contributions

Each author has contributed to the submitted work as follows: VK, P-YS, and RA are the guarantors of the paper. PT, DB, and RA designed the study. PT and PC-R realized statistics. RA, NK, and PR analyzed the data. PT and RA drafted the manuscript. NK, PR, P-YR, and PC-R revised the manuscript. All authors contributed in drawing up the manuscript.

## Conflict of Interest Statement

The authors declare that the research was conducted in the absence of any commercial or financial relationships that could be construed as a potential conflict of interest. The reviewer, PH, and handling Editor declared their shared affiliation.
